# Comparative Demography of the Spider Mite, *Oligonychus afrasiaticus,* on four Date Palm Varieties in Southwestern Tunisia

**DOI:** 10.1673/031.011.13601

**Published:** 2011-10-13

**Authors:** Sameh Ben Chaaban, Brahim Chermiti, Serge Kreiter

**Affiliations:** ^1^ Institut Supérieur des Sciences Agronomiques de Chott-Mariam, Département de Protection des Plantes, Laboratoire de Zoologie Agricole, 4042 Sousse, Tunisia; ^2^ Montpellier SupAgro, UMR CBGP, bâtiment 16, 2 Place Pierre Viala, 34060 Montpellier cedex 01, France

**Keywords:** Acari, Deglet Noor, life table parameters, Tetranychidae

## Abstract

The date palm mite, *Oligonychus afrasiaticus* (McGregor) (Acari: Tetranychidae), is a serious pest of palm date fruits. Life cycle, fecundity, and longevity of this mite were studied on fruits of four date palms, *Phoenix dactylifera* L. (Arecales: Arecaceae)(varieties: Deglet Noor, Alig, Kentichi, and Besser), under laboratory conditions at 27 = 1 ^°^C, 60 ± 10% RH. Total development time of immature female was shorter on Deglet Noor *fruits* than on the other cultivars. *O. afrasiaticus* on Deglet Noor had the highest total fecundity per female, while low fecundity values occurred on Besser. The comparison of intrinsic rates of natural increase (*r_m_*), net reproductive rates (*R_o_*), and the survival rates of immature stage of *O. afrasiaticus* on the host plants suggests that *O. afrasiaticus* performs better on Deglet Noor fruits. The mite feeding on Alig showed the lowest intrinsic rate of natural population increase (*r_m_* = 0.103 day ^1^). The estimation of difference in susceptibility of cultivars to *O. afrasiaticus* is crucial for developing efficient pest control programs. Indeed, less susceptible cultivars can either be left unsprayed or sprayed at low threshold.

## Introduction

In the southwestern region of Tunisia, the date palm, *Phoenix dactylifera* L. (Arecales: Arecaceae), is often prone to phytophagous attacks caused by various insects, particularly in areas like Djérid (Gouvernorat of Tozeur) and Nefzaoua (Gouvernorat of Kebili), where date production is high. The three principal species threatening date production are the carob moth *Ectomyeloïs ceratoniae*, the white scale *Parlatoria blanchardi*, the beetle *Oryctes agamemnon*, and the acarina Boufaroua *Oligonychus* afrasiaticus (McGregor) (Acari: Tetranychidae). The latter is a serious pest of date palm fruits in Tunisia (Dhouibi 1991; Khoualdia et al. 1997).

The infestation of *O. afrasiaticus* begins in and increases throughout the summer months during the fruit's kimri stage, characterized by greenness. Feeding on the immature dates causes severe fruit scarring, sometimes to such a degree that the dates turn brown and have a scabbed appearance. Such damages have caused reductions in fruit grade and subsequent crop losses. In Algeria in 1981, 30–70% of dates were discarded (Guessoum 1986). In Mauritania, annual production was reported as “unmarketable” on 70% of infested trees (De Montaigne and Mouloud 1986). Typically, mite populations begin to decline when fruits change to yellow or red at the khalaal stage.

Date palm varieties in several areas have shown varying degrees of susceptibility to *O. afrasiaticus.* In the Gulf area, Hussain (1974) indicated that the Iraqi variety ‘Sayer’ was relatively resistant to mite attacks. In Saudi Arabia, date fruit cultivars ‘Sokary’ and ‘Roman’ appeared to be highly susceptible to *O. afrasiaticus,* contrary to ‘Cebiky’ cultivar that seemed to be resistant, and to the ‘Khodary’ cultivar that appeared to be moderately resistant to attacks by this mite species (Aldosari and Ali 2007). In Oman, the cultivars ‘Hilali’, ‘Gibri’, and ‘Khanazani’ were infested by *O. afrasiaticus* during the month of April, whereas other cultivars were attacked later in the growing season (Elwan 2000). In Israel, Palevsky (2005) reported that the ‘Deglet Noor’ cultivar was more targeted than both the ‘Medjool’ and ‘Barhi’ cultivars. In north Africa, more specifically in Libya, the varieties ‘Asabir’, ‘Aurig’, ‘Bestian’, ‘Apel’, and ‘Talise’ were found to be more attractive and susceptible to this mite than ‘Tafsirt’, which was found to be less susceptible (Edongali et al. 1988). In a previous study conducted in the southwestern region of Tunisia, it was found that mite phenology was affected by date palm cultivar (Ben Chaaban and Chermiti 2010). Pest populations increased first on Deglet Noor and later on Besser cultivar. Population levels fluctuated from year to year and were usually lower on Kentichi and Besser than on Alig and Deglet Noor cultivars.

The present study was designed to provide data on developmental rate and fecundity of a local population of *O. afrasiaticus* at a constant temperature in the laboratory on fruits of different palm date varieties. This knowledge may prove useful for identifying susceptible and resistant varieties.

## Materials and Methods

### Mite cultures

Date palm fruits (var. Deglet Noor) that were highly infested with *O. afrasiaticus* were collected from the Segdoud oases, located near Tozeur, Southern Tunisia, in July 2006. After several hundred spider mites of all stages were collected, a stock colony was started on sorghum plants (*Sorghum* sp.).

One month later, hundreds of females and males were randomly selected from the laboratory culture and were placed together on rearing units with dates varieties Alig, Besser, Kentichi, and Deglet Noor to lay eggs. Eggs were conserved until they reached maturity, and were used for experiments in this study. This was done to ensure that mites used in experiments were produced from food with a known rate of increase (*r_m_*). The spider mite populations in the colonies were never below several hundred individuals.

All colonies, were kept in a room at 25 ± 1° C, 62.5 ± 12.5 % RH, and a 16:8 h (L:D) photoperiod.

### Estimation of *O. afrasiaticus* developmental time

*O. afrasiaticus* juvenile survival and developmental time of males and females were determined when feeding on fruits of Deglet Noor, Alig, Kentichi, and Besser varieties. Dates were collected during the period of kimri stage.

All experiments were conducted in a climate-controlled room at 27 ± 1° C, 60 ± 10 % RH, and a photoperiod of 16:8 L:D. Fruits were placed on a water-saturated foam mat in a plastic tray. Water-saturated cotton wool was used to prevent mite escape and maintain leaf freshness. The cotton wool was kept wet by periodically adding distilled water.

A few days prior to the starting of the test, 50 deutonymphal (2^nd^ nymphal stage) females were randomly selected from the corresponding colony and held separately on each date. To ensure mating, two adult males were placed with each newly emerged adult female. After two days, 10 mated females were placed together on five dates to lay eggs. An hour later, all females were eliminated and eggs were counted. This operation was repeated until 100 eggs were obtained. Before hatching, eggs were observed at six-hour intervals. After hatching, larvae were observations daily from 06:00 and 18:00 until they reached maturity. Larvae were reared on fresh date fruits.

### Estimation of *O. afrasiaticus* reproductive parameters and longevity

Pre-oviposition, oviposition, and post-oviposition periods, total fecundity, daily oviposition rate, and female longevity were determined on each host plant. For each host plant, 50 deutonymphal females were randomly selected from the corresponding colony. To ensure mating, two adult males were placed with each newly emerged adult female on the date fruit. The number of eggs deposited by each female was recorded daily until all females died. The eggs obtained from each female were cultured to determine their hatchability. Pre-oviposition period was recorded at six-hour intervals, while other reproductive parameters and longevity were recorded every 12 hours. Dates were removed and replaced at two-day intervals.

### Sex ratio

Sex ratio is described as the proportion of females in the progeny. We evaluated sex ratio of twenty *O. afrasiaticus* females for each host plant. The method was the same as that used for oviposition, except that females were placed on a new fruit every day, and dates with eggs were maintained under the same experimental conditions as for females. The sex ratio was determined on the basis of a count of adults originating from those eggs. Because *O. afrasiaticus* exhibits arrhenotokous parthenogenesis (i.e., unfertilized eggs only produce haploid males), unmated females were not taken into account.

**Figure 1.**
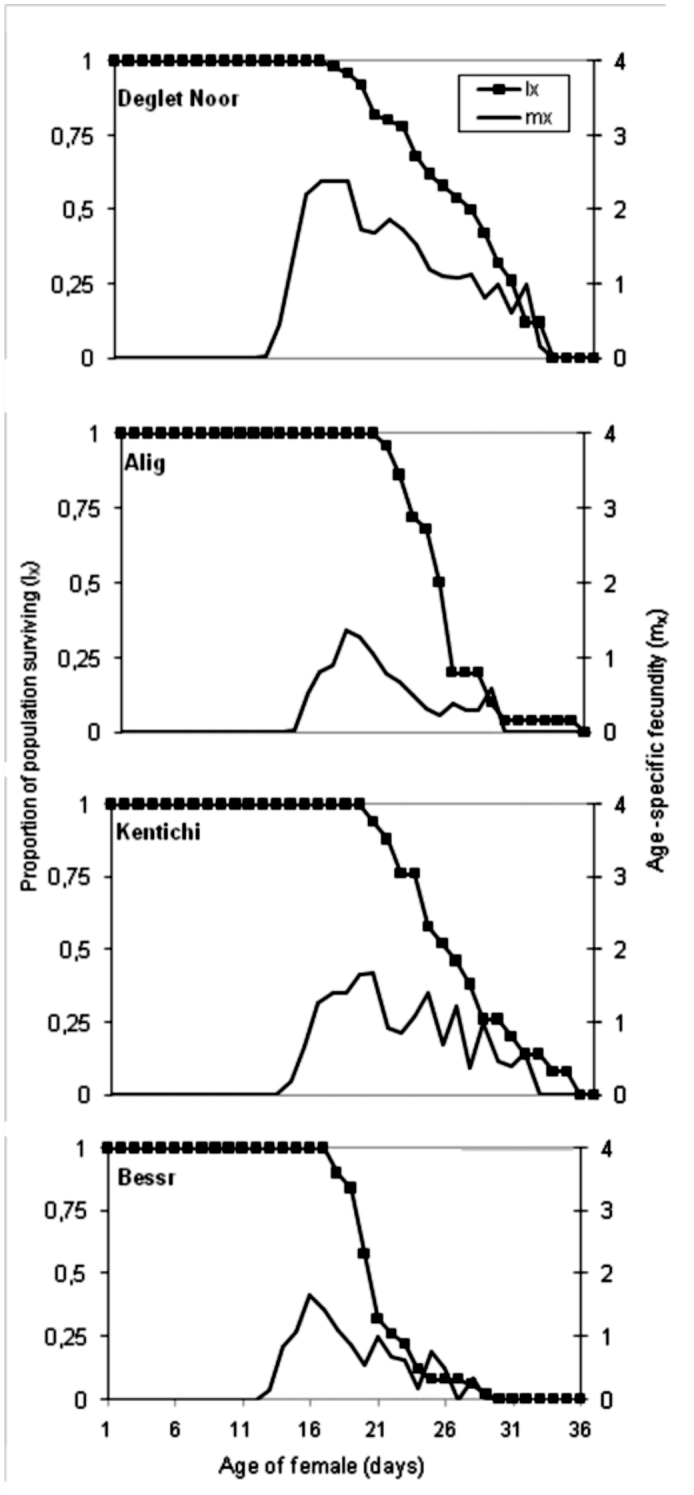
Survivorship curve (lx) and age-specific fecundity (m_x_) of *Oligonychus afrasiaticus* on four date palm varieties at 27 ^°^C. High quality figures are available online.

### Life tables

The life table was constructed considering the female cohort studied in this experiment. The net reproductive rate (*R_o_*, mean number of female progeny produced by a single female during its mean lifetime, expressed in♀/♀); gross reproductive rate (*GRR,* in ♀/♀), generation time (*T,* mean period between birth of the parents and that of the offspring, measured in days), intrinsic rate of increase (*r*, ♀/♀/days), finite rate of increase (λ, in ♀/♀/days), and doubling time (*D_t_*, time for population to double, measured in days) were calculated using the method recommended by Birch (1948).

### Statistical analysis

Data on developmental time, duration of female reproductive periods, and fecundity were analyzed using one-way ANOVA followed by Tukey's test (*p* = 0.01) to compare data means. Differences in sex ratio were analyzed by a Chi-square (χ^2^) test. SPSS 10 (Statistical Package for the Social Sciences, version 10) was used for statistical analysis.

## Results

### Developmental time of immature stages

The duration of the whole immature phase (egg to adult emergence) varied significantly between host plants. Development duration was categorized as low on Deglet Noor, high on Alig, and intermediate on Kentichi and Besser cultivars (Tukey's test, *p* < 0.01, Table 1). For developmental duration in males, statistical analysis showed significant differences among host plants (Tukey's test, *p* < 0.01, Table 1); the highest means of the development period were registered on Kentichi and Alig cultivars, while the lowest mean registered on Besser and Deglet Noor cultivars. The total development duration was significantly longer for females than for males on all host plants (Tukey's test, *p* < 0.01, Table 1).

**Table 1.**
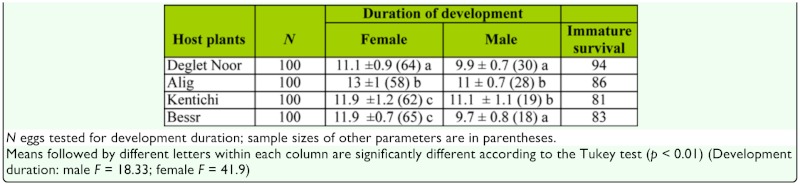
Means (± SD) in days of development duration of females and males and immature survival of *Oligonychus afrasiaticus* at 27 °C in four date palm cultivars.

**Table 2.**
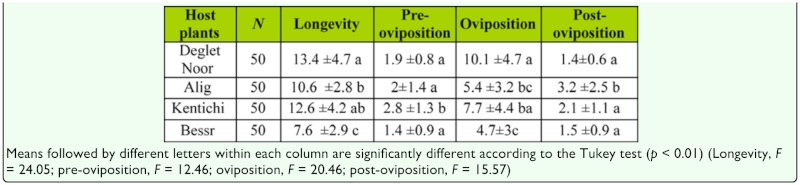
Adult longevity, pre-oviposition, oviposition, and post-oviposition times (± SD) (days) of *Oligonychus afrasiaticus* at 27 °C in four date palm cultivars. Number of replicates (*N*).

### Adult female longevity

The longevity of adult female *O. afrasiaticus* and the length of the pre-oviposition, oviposition, and post-oviposition periods differed significantly between the four host plants. The longest pre-oviposition and post-oviposition periods were registered on Kentichi and Alig, respectively (Table 2, *p* < 0.01). The oviposition period was longer on Deglet Noor fruits than on other hosts (Table *2, p* < 0.01). Deglet Noor fruits had the highest longevity, while the lowest longevity was recorded on Besser fruits (Table 2, *p* < 0.01).

### Fecundity and hatchability

The total number of eggs laid per female was highest on Deglet Noor fruits and lowest on Besser fruits (Table 3, *p* < 0.01). Daily egg production obtained on Alig, Kentichi, and Besser fruits was less than one egg per female, while egg production on Deglet Noor fruits was 1.5 eggs/female. The peak of this parameter was reached on day 18 (1.36 eggs/female/day), day 16 (1.66 eggs/female/day), day 18 (2.4 eggs/female/day), and day 20 (1.7 eggs/female/day) on Alig, Besser, Deglet Noor, and Kentichi, respectively. Thereafter, egg production decreased gradually (Figure 1).

In general, there was no distinct peak for maximum production; egg production on all tested varieties was distributed over a relatively long time period, and survival declined gradually after an extended oviposition period. No significant difference in hatchability was observed between the different plant-based food resources. The lowest hatchability was observed with Kentichi dates (Table 3).

### Sex ratio

There were no significant effects of plant-based foods on sex ratio of the descendant of *O. afrasiaticus* (Table 3; χ^2^, *p* > 0.05). The sex ratio was always biased toward females.

**Table 3.**
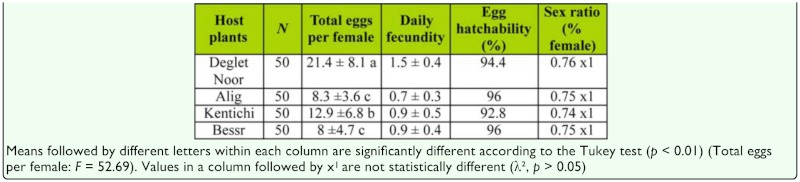
Number of eggs per female, daily fecundity (eggs/female/day), egg hatchability, and sex ratio of *Oligonychus afrasiaticus* at 27 °C in four date palm cultivars.

**Table 4.**
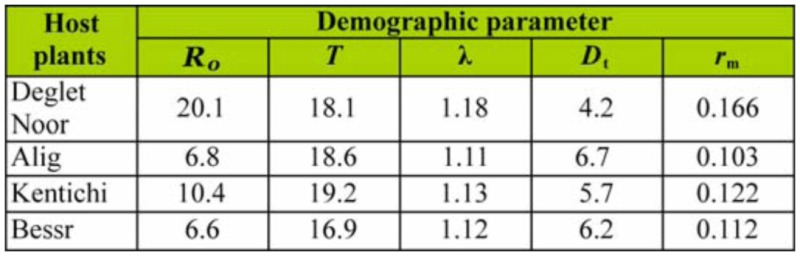
Demographic parameters of *Oligonychus afrasiaticus* at 27 °C in four date palm cultivars: net reproductive rate (*R_o_*), mean generation time (*T*), intrinsic rate of increase (*r*m), doubling time (*D*t), and finite rate of increase (λ).

### Life table

Life table parameters are presented in Table 4. These results showed that parameters such as intrinsic rate of natural increase (*r_m_*), net reproductive rate (*R_o_*), doubling time (*D_t_*) of *O. afrasiaticus* differed between the for host plants.

The longest mean generation time (*T*) occurred on Kentichi, followed by Alig, whereas the shortest mean generation time was on Besser. Net reproductive rate (*R_o_*) was highest on Deglet Noor fruits (20.1 offspring/female). Along with the lowest observed duration of development and higher rates of oviposition, mites reared on Deglet Noor fruits showed the highest value of intrinsic rate of natural increase (*r_m_* = 0.166 day-^1^), while mites on Alig had the lowest intrinsic rate of increase (*r_m_* = 0.103 day^-1^). Consequently, feeding on Deglet Noor fruits engenders the shortest mite doubling time (*D_t_ =* 4.2 days).

## Discussion

Our study shows that *O. afrasiaticus* feeds, survives, and develops on all four date palm cultivars, and that host type can greatly affect *O. afrasiaticus* development, fecundity, and life-table parameters. Indeed, the results showed that the host plant had substantial effects on intrinsic rate of natural increase (*r_m_*), net reproductive rate (*R_o_*), and survival of the adult stage.

Developmental times reported in this study on the four cultivars were significantly different. Gutierrez (1977) found that the total duration of the immature stages of *O. thelytokus* development, with temperature at 25 °C and relative humidity between 60 and 70%, was 13.3 days. Previous studies had demonstrated cultivar effects on life cycle and population increase of a variety of Tetranychid species. For example, the life cycle of *O. punicae* differed among grape cultivars with average values ranging between 8.2 days on Tucupita leaves to 9.1 days on Sirah (Vásquez et al. 2008). Another example is the developmental time of *Amphitetranychus viennensis,* which was found to be lower when reared on apple cultivars ‘Starkrimson Delicious’ and ‘Golden Delicious’ (10.7 days) than on ‘Amasya’ and ‘Starking Delicious’ (11.7 days) (Kasap 2003).

Also, female longevity of *O. afrasiaticus* was highly affected by the nature of the host plant. The longest period was registered on Deglet Noor fruits, while the shortest period was found on Besser fruits. Vásquez et al. (2008) found that mean longevity of *O. punicae* was affected by grape cultivar; females lived longest on ‘Sauvignon’ (17.5 days) and shortest on ‘Villanueva’ (8.1 days). The mean *O. perseae* female longevity increased in the susceptible avocado cultivar ‘Hass’ by 100%, from 12 days in May to 24 days in July. However, more resistant cultivars such as ‘Pinkerton’ and ‘Lamb Hass’ showed a 30% drop in longevity over the same period (Kerguelen and Hoddle 2000).

Total fecundity was higher on Deglet Noor fruits than on the other cultivars. An effect of host plant on reproduction has been established for several Tetranychid species (e.g., Ribeiro et al. 1988; Krips et al. 1998; Kerguelen and Hoddle 2000; Hilker and Meiners 2002; Vásquez et al. 2008; Razmjou et al. 2009).

The rate of immature survival ranged from 81– 94 % with the highest registered rate on Deglet Noor fruits. Similarly, Kerguelen and Hoddle (2000) found that *O. perseae* survival was significantly different across three avocado cultivars.

The *r_m_* value of *O. afrasiaticus* estimated in the current study ranged from 0.166 to 0.103 individuals per female per day. The *r_m_* values of *Oligonychus* mites varied between 0.178 and 0.290 day-^1^ at ∼ 25° C (Perring et al. 1984; Saito 1979). Perhaps the chemical content and hardness of the date fruit exocarp are responsible for the low *r_m_* of *O. afrasiaticus* on date fruit cultivars. However, our results are close to those estimated for other *Oligonychus* mites. For example, *O. punicae* studied on six grapevine cultivars at 27 ± 2° C showed an *r_m_* that varied between 0.292 and 0.135 (Vásquez et al. 2008). The *r_m_*value calculated by Gutierrez (1977) for *O. thelytokus* was 0.115 individuals per female per day.

The intrinsic rate of natural increase (*r_m_*) is an important parameter to describe population growth potential under specific climatic and food conditions, because it reflects the overall effects of temperature and food on development, reproduction, and survival (Southwood 1978). Two parameters of paramount importance in determining the *r_m_*value are developmental time and oviposition rate (Helle and Sabelis 1985). Therefore, Alig cultivar proved to be the least suitable host for *O. afrasiaticus.* This was not only reflected in fecundity, but also in the slower development times. In contrast, *O. afrasiaticus* has the best performance on Deglet Noor fruits. This was mainly due to a short development time, high daily egg production, an early reproduction peak, and higher survivorship of immature developmental stages. The mite exhibited an intermediate population increase when fed on Kentichi and Besser cultivars. Differences in development, reproduction, fertility, longevity, and population development of tetranychid mites on different host plants are common. These differences may be associated with impediments to feeding such as host plant texture, nutritional value of the host, and host physiology (Bengston 1970; Helle and Sabelis 1985; Archer et al. 1986; Kielkiewicz and Van de Vrie 1990; Kerguelen and Hoddle 2000; Kasap 2003; Ragusa and Farragut 2005; Kafil et al. 2007; Vásquez et al. 2008).

Recent studies have demonstrated that the hardness of the date fruit exocarp does not appear to be a factor affecting *O. afrasiaticus* establishment, because mite populations were highest on Deglet Noor cultivar in July when resistance to penetration was greater than on Alig and Besser cultivars (Ben Chaaban & Chermiti 2009). Also, Palevsky (2005) found higher densities of *O. afrasiaticus* on date cultivars ‘Medjool’, ‘Barhi’, and ‘Deglet Noor’ in July despite the fruit hardness. Studies investigating the influence of leaf anatomy on tetranychid life-history parameters have not been conclusive. Skorupska (1998) showed that stoma count on the abaxial surface, spongy and palisade mesophyll, and total leaf lamina width affected growth of *Amphitetranychus viennensis* in apple cultivars. Conversely, Nukenine et al. (2000) did not find any relationship between anatomical features in cassava and resistance to the cassava green mite. Similarly, foliar anatomy of grape cultivars did not appear to hinder *O. punicae* feeding, because mites reached adulthood also on cultivars Red Globe and Sauvignon despite greater cuticle-epidermis thickness (Vásquez et al. 2008).

The performance of *O. afrasiaticus* varied greatly between cultivars depending on water content, sugar levels, proteins content, and acidity (Palevsky et al. 2005; Aldosari and Ali 2007; Ben Chaaban and Chermiti 2009). In Tunisian oases, a study of the population dynamics of *O. afrasiaticus* on Deglet Noor, Alig, and Besser cultivars in relation to date fruit chemical showed that Deglet Noor was most suitable. Mites were detected earlier in Deglet Noor than in the two other cultivars. The fruit acidity was similar in the three cultivars early in the growing season. Deglet Noor fruits became particularly favorable to *O. afrasiaticus* reproduction when the fruit acidity decreased from 2.2 to 0.4 mEq/100 g. However, Besser and Alig cultivars could be resistant to *O. afrasiaticus* because of higher acidity (0.8 mEq/100 g) (Ben Chaaban and Chermiti 2009).

Sugars are known to act as phagostimulants in insects (Bernays 1985) and mites (Rodriguez and Rodriguez 1987). Several studies have demonstrated a positive correlation between mite population growth and leaf sugar concentration of several host plants (Rodriguez et al. 1960; Rodriguez and Cambell 1961). However, sugar levels could be a limiting factor for mite settling. For example, young leaves of Gerbbera seem more resistant to *Tetranychus urticae* than mature leaves because of its higher content of reducing sugars (Kielkiewicz 1995). Similarly, carbohydrates play an important role in the development of *O. afrasiaticus,* but could also be a source of resistance used by plants against mites (Palevsky et al. 2005). Early in the season in Tunisian oases, the restricted sugar level on fruits prevented *O. afrasiaticus* establishment on Deglet Noor, Alig, and Besser fruits. Mites were first detected in Deglet Noor fruits because reducing sugar levels increased earlier than in the two other cultivars. In contrast, Besser infestation was late, suggesting the restricted sugar level could act to provide plant resistance. Population decline did coincide with an increase in total sugar levels in all three cultivars (Ben Chaaban and Chermiti 2009).

Analysis indicated that the decrease of this pest was positively correlated with the total soluble solids values and negatively with the water content (Palevsky et al. 2005; Ben Chaaban and Chermiti 2009).

This study shows that population density and fecundity of *O. afrasiaticus* are dependent on host-plant quality. Demographic parameters of this mite pest are better on *Deglet Noor cultivar* than on *Besser, Alig, and Kentichi cultivars,* suggesting that *Deglet Noor* is more suitable for *O. afrasiaticus* than *the other cultivars.* Identifying differences in susceptibility to *O. afrasiaticus* is crucial for developing effective pest control programs. Varieties of date palm that are less susceptible can be left unsprayed or sprayed at a very low threshold. Increasing variety diversity in orchards should be considered an important strategy in reducing damage and associated yield losses caused by *O. afrasiaticus.* Further research must be performed to elucidate the nature of host suitability.
